# Digital Health Rights, Adult Systems: Are We Leaving Adolescents Behind?

**DOI:** 10.1016/j.mcpdig.2025.100254

**Published:** 2025-07-22

**Authors:** Louisa Peralta, Cristyn Davies, Kellie Burns, Leena Paakkari

**Affiliations:** a Sydney School of Education and Social Work, The University of Sydney, New South Wales, Australia; b Speciality of Child and Adolescent Health, Faculty of Medicine and Health, The University of Sydney, New South Wales, Australia; c School of Social Sciences, Western Sydney University, New South Wales, Australia; d The Faculty of Sport and Health Sciences, The University of Jyväskylä, Finland

The digital transformation of health care is reshaping access to services and health information, emerging as a significant determinant of health.^1^ One key element of this shift is the patient portal—an interface that allows individuals to access their electronic health records and communicate with health care providers. These portals hold promise for improving care access, fostering health awareness, supporting self-management, and encouraging patient engagement.^2^^,^^3^ However, meaningful use of these tools relies heavily on individuals’ health and digital literacy levels.^4^

As patient rights expand, these developments increasingly impact adolescents. In many high-income countries—including in Europe—adolescents as young as 12-13 years can access their electronic health records and exercise control over who sees their information.^5^ This includes the right to restrict parental access. As adolescents gain rights to use digital health services and make independent health decisions, enhancing their health literacy capabilities is imperative to ensure that they can navigate these systems effectively. However, the question remains: Are we adequately preparing adolescents for this role?

Health literacy—the ability to find, understand, appraise, and apply health information^6^—has been linked to increased and effective use of patient portals.^4^^,^^6^ Adolescents with low health literacy may struggle to interpret clinical information, assess benefits and risks, or make informed health decisions. Limited critical reading skills can result in misinterpretation of medical data, potentially leading to inaction, unhealthy behaviors, or inappropriate self-treatment. Moreover, early exposure to sensitive clinical information—often presented in highly technical language—can overwhelm young users. There is growing concern that such access, when unsupported, may do more harm than good.^7^

Adolescents are not a homogeneous group in terms of digital competence. Although some are digitally fluent and can leverage online tools for health promotion, others lack the necessary skills to evaluate online information, protect their privacy, or use health portals effectively.^1^ This digital divide exacerbates health inequities, especially when digital tools are assumed to be universally accessible and usable for all young people.

A commonly proposed solution is shared access between adolescents and their parents. This model, used in many countries, allows the parents to support the navigation of patient portals. However, shared access raises ethical concerns. Adolescents may consider certain health information—such as substance use, sexual activity, or mental health—as private. Fear of parental judgment may deter them from disclosing important information to health care providers, undermining trust and care quality.^7^ Empowering adolescents must therefore go beyond offering access. It requires a dual focus on building individual capacity and creating supportive system-level design.

Although global health initiatives have promoted health literacy development through school-based education, major gaps remain—not only in adolescents’ health literacy levels,^8^ but also in teachers' ability to deliver effective health education.^9^ Many school policies still lack a clear framework for implementing health literacy programs. Even where frameworks exist, evaluations of their effectiveness are rare. Stronger partnerships across health, education, and youth stakeholders are needed to codesign not only research, education, and policy that meet adolescents’ health literacy needs but also their cognition, values, and goals for their own health.^7^

Furthermore, current health portals are largely built for adult users, often overlooking the developmental needs of adolescents. Without intentional design that considers young users’ literacy levels, cognitive development, and privacy needs, we risk placing unfair expectations on adolescents while offering inadequate support. This raises a critical ethical issue: if systems continue to evolve without integrating adolescent-centered design and responsiveness, we may hold adolescents accountable as informed health consumers without enabling them to succeed. As Psihogios et al^7^ aptly note, “adolescents are still waiting on a digital health revolution.”

Such a revolution would involve redesigning portals to be user-friendly—using age-appropriate or plain language, simplified navigation, and built-in educational tools such as glossaries, explainer videos, or interactive content.^10^ These design elements could help bridge the gap between access and understanding, ensuring that adolescents can not only view their records but also make sense of them and act accordingly.

In summary, although patient portals offer significant opportunities for adolescent engagement in health care, they also pose risks when access outpaces capability. Adolescents’ limited health and digital literacy, coupled with systems designed primarily for adults, can inadvertently widen rather than reduce health disparities. Efforts to make portals more accessible must go hand-in-hand with investments in education and system redesign. Only through a coordinated approach that promotes adolescent health literacy and health system responsiveness can we truly empower young people to take charge of their health in the digital age.

## Potential Competing Interests

The authors report no competing interests.
